# Supplementary material for: Asymmetric Synthesis of Quaternary Hydantoins via a Palladium-Catalyzed Aza-Heck Cyclization
Source: J Am Chem Soc. 2025 Nov 14;147(49):44692–8. doi: 10.1021/jacs.5c16022 (PMC12703750; doi:10.1021/jacs.5c16022)
Supplement: Supplementary file 2 [file ja5c16022_si_002.zip › All NMR FID Files/S41/S41_AllNMR/1/pdata/1/email_EM04-042-dry_1_1.pdf]

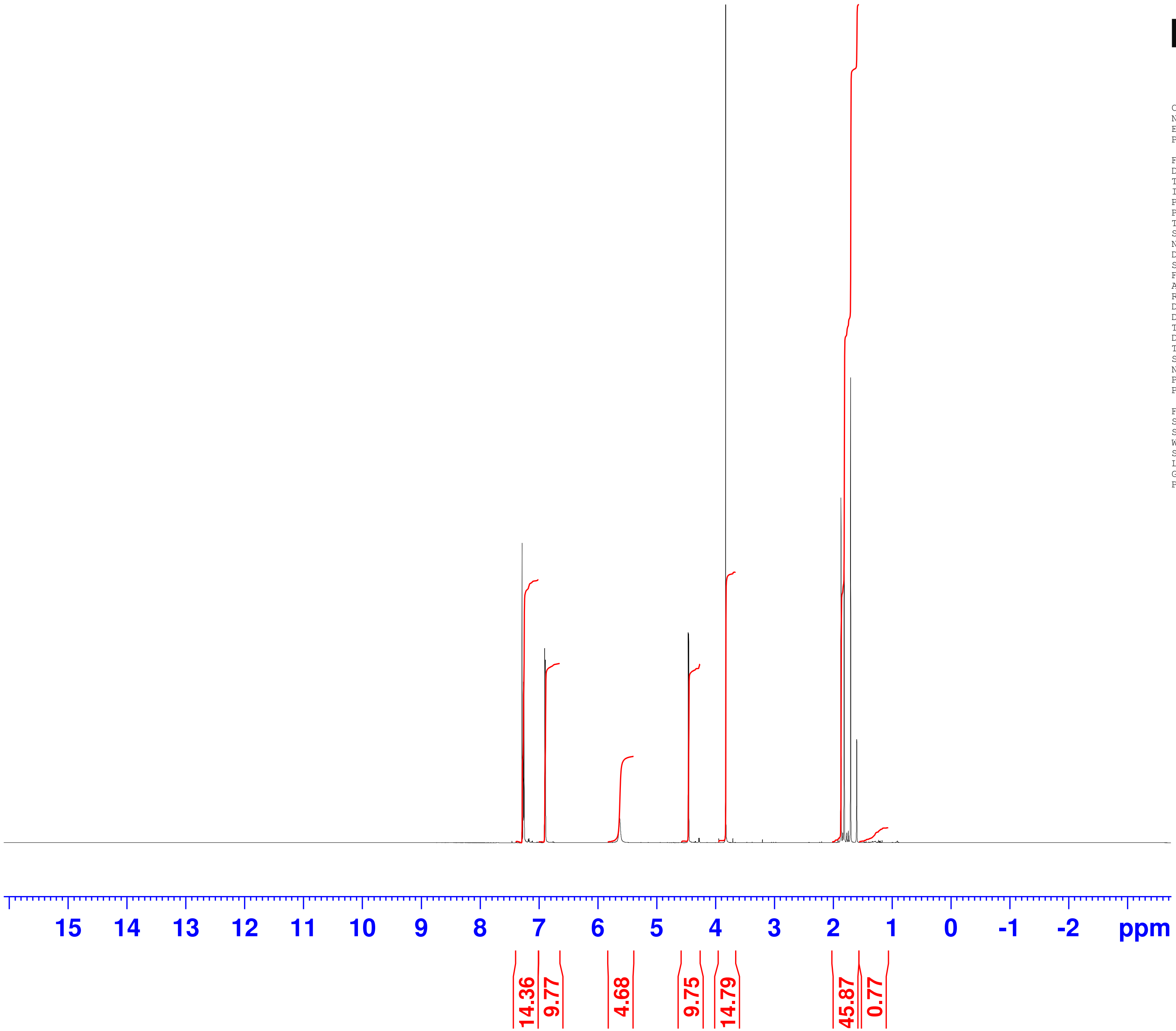

Current Data Parameters

NAME	EM04-042-dry	
EXPNO	1	
PROCNO	1	

F2 - Acquisition Parameters

Date_	20231006	
Time	11.47	h
INSTRUM	Avance Neo 600	at University of Delaware
PROBHD	Z185170_0001 (	
PULPROG	zg	
TD	65536	
SOLVENT	CDCl3	
NS	16	
DS	2	
SWH	11904.762	Hz
FIDRES	0.363304	Hz
AQ	2.7525120	sec
RG	12.7	
DW	42.000	usec
DE	10.65	usec
TE	298.0	K
D1	10.00000000	sec
TD0	1	
SFO1	600.1337058	MHz
NUC1	1H	
P1	8.01	usec
PLW1	6.90000010	W

F2 - Processing parameters

SI	65536	
SF	600.1300000	MHz
WDW	EM	
SSB	0	
LB	0.30	Hz
GB	0	
PC	1.00	
